# Clinical usefulness of early serial measurements of C-reactive protein as outcome predictors in patients with subarachnoid hemorrhage

**DOI:** 10.1186/s12883-020-01687-3

**Published:** 2020-03-27

**Authors:** Sangkil Lee, Yong Oh Kim, Jeong-Am Ryu

**Affiliations:** 1 Department of Neurology, ChungBuk National University Hospital, Cheongju, Republic of Korea; 2 Department of Critical Care Medicine, Samsung Medical Center, Sungkyunkwan University School of Medicine, 81 Irwon-ro, Gangnam-gu, Seoul, 06351 Republic of Korea; 3 Department of Neurosurgery, Samsung Medical Center, Sungkyunkwan University School of Medicine, 81 Irwon-ro, Gangnam-gu, Seoul, 06351 Republic of Korea

**Keywords:** C-reactive protein, Prognosis, Subarachnoid hemorrhage, Vasospasm, Inflammation

## Abstract

**Background:**

The purpose of this study was to evaluate the role of C-reactive protein (CRP) in predicting neurological outcomes of patients with subarachnoid hemorrhage (SAH).

**Methods:**

In this retrospective, observational study of adult patients with SAH treated between January 2012 and June 2017. Initial CRP levels collected within 24 h from the onset of SAH, the follow-up CRP levels were measured. The primary outcome was neurological status at six-month follow-up assessed with the Glasgow Outcome Scale (GOS, 1 to 5).

**Results:**

Among 156 patients with SAH, 145 (92.9%) survived until discharge. Of these survivors, 109 (69.9%) manifested favorable neurological outcomes (GOS of 4 or 5). Initial CRP levels on admission and maximal CRP levels within four days were significantly higher in the group with poor neurological outcome compared with those manifesting favorable neurological outcomes (*P* = 0.022, *P* < 0.001, respectively). However, the clearance of CRPs did not differ significantly between the two groups (*P* = 0.785). Analysis of the receiver operating characteristic curve for prediction of poor neurological outcome showed that the performance of the maximal CRP was significantly better compared with the initial CRP or the clearance of CRP (*P* = 0.007, *P* < 0.001, respectively). In this study, the effect of CRP on neurological outcomes differed according to surgical clipping. The maximal CRP levels within four days facilitate the prediction of neurological outcomes of SAH patients without surgical clipping (C-statistic: 0.856, 95% confidence interval [CI]: 0.767–0.921). However, they were poorly associated with neurological prognoses in SAH patients who underwent surgical clipping (C-statistic: 0.562, 95% CI: 0.399–0.716). Multivariable logistic regression analysis revealed that age (adjusted odds ratio [OR]: 1.10, 95% CI: 1.052–1.158), initial Glasgow Coma Scale (adjusted OR: 0.74, 95% CI: 0.647–0.837), and maximal CRP without surgical clipping (adjusted OR: 1.27, 95% CI: 1.066–1.516) were significantly associated with poor neurological outcomes in SAH patients.

**Conclusions:**

Early serial measurements of CRP may be used to predict neurological outcomes of SAH patients. Furthermore, maximal CRP levels within four days post-SAH are significantly correlated with poor neurological outcomes.

## Background

Subarachnoid hemorrhage (SAH) is a disease associated with high mortality. Survivors of SAH may manifest severe neurological deficits [1–4]. In patients with SAH, early brain injury or delayed cerebral ischemia (DCI) is associated with poor outcomes [1, 5, 6]. Although the pathogenesis of DCI has been poorly understood [1, 5–7], activated inflammation after SAH could be one of the most critical factors affecting the development of DCI [5, 8].

Generally, C-reactive protein (CRP) is a useful marker of non-specific inflammation [8, 9]. Elevated CRP levels might be associated with the progression of vascular disease [8, 9]. Elevated levels of CRP are significantly associated with unfavorable long-term functional outcome in patients with ischemic stroke [9, 10]. Additionally, the measurement of CRP significantly increases the ability to make accurate predictions and prevent or manage coronary thrombotic events appropriately [11–13]. Recent studies suggest that elevated CRP levels are associated with vasospasm and DCI after SAH [11, 13–15]. Several inflammatory mechanisms directly mediate the pathogenesis of cerebral vasospasm. DCI with increased soluble adhesion molecules and cytokines, which are associated with the pathogenesis of cerebral vasospasm, also strongly stimulates CRP synthesis [13]. Therefore, the measurement of CRP concentrations may facilitate the prediction of clinical outcomes in SAH patients. Limited studies have reported that the elevated baseline CRP might be associated with the prognosis of SAH patients [11, 16]. However, it is not established whether the initial/maximal CRP levels or changes in their levels predict the neurological outcomes in SAH patients. In addition, several studies have shown that CRP measurements at different time points are associated with the prognosis of patients with ischemic stroke [9, 10, 17, 18]. Therefore, the objective of this study was to investigate if CRP levels or changes in their levels may be used to predict neurological outcomes of patients with SAH, and the time points of CRP measurements that are most relevant to predict the prognosis of patients with SAH.

## Methods

### Study population

**Fig. 1:**
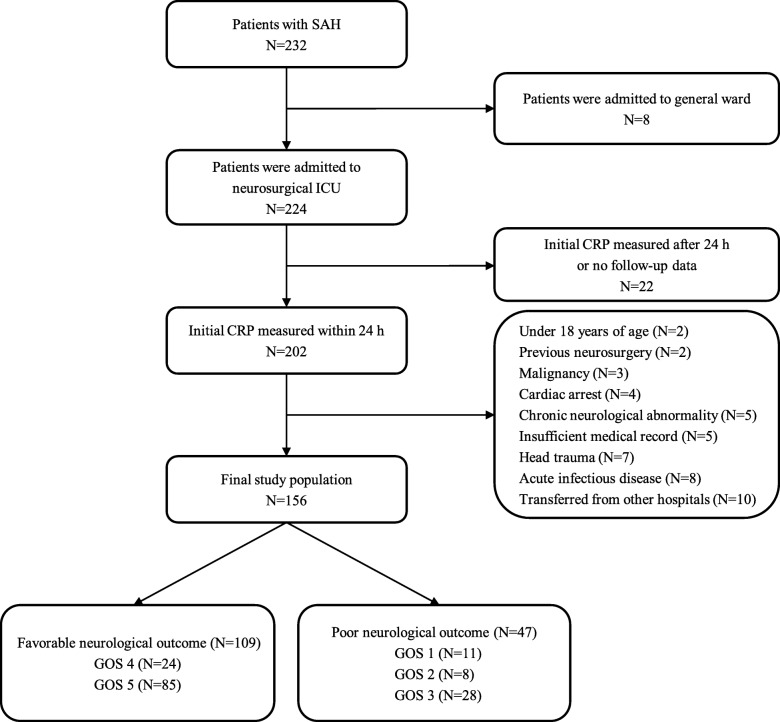
Study flow chart. SAH, subarachnoid hemorrhage; ICU, intensive care unit; CRP, C-reactive protein; GOS, Glasgow Outcome Scale

This investigation was a retrospective, single-center, observational study of adult patients with SAH who were admitted to the neurosurgical intensive care unit (ICU) at Samsung Medical Center (SMC) from January 2012 through June 2017. This study was approved by the Institutional Review Board of SMC (SMC 2018–07-154). The requirement for informed consent was waived due to its retrospective study design. This study was conducted using the SMC SAH registry in the same way as our previous study [4]. In this study, brain computed tomography (CT) scan was routinely performed to evaluate the causes of severe and sudden onset headache or unconsciousness. If SAH was detected or suspected during the initial brain CT scan, CT angiography was immediately performed to diagnose SAH. Patients with Fisher grade > 1 in initial brain CT or CT angiography and Hunt-Hess grade > 1 on admission were admitted to neurosurgical ICU, and those with initial CRP levels collected within 24 h from the onset of SAH and with follow-up CRP levels were included. Of these patients, those who were aged below 18 years, those diagnosed with malignancy and an expected life span of less than one year, those with a history of head trauma, neurosurgery, cardiac arrest, or chronic neurological abnormality on admission, those with insufficient medical records, and those who were transferred from other hospitals after more than one day of onset of SAH were excluded [4]. Patients diagnosed with an acute infectious disease were also excluded. Finally, a total of 156 patients diagnosed with SAH were analyzed in this study (Fig. 1).

### Endpoints and definitions

In this study, poor-grade SAH was defined as World Federation of Neurosurgical Societies (WFNS) grade 4 or 5 [1]. The primary endpoint was poor neurological outcome at six months after the admission. The neurological status was assessed by the Glasgow Outcome Scale (GOS, 1 to 5) [4, 19]. In this study, GOSs of 4 and 5 were classified as favorable neurological outcomes, whereas GOSs of 1, 2, and 3 were considered as poor neurological outcomes. Medical records of patients were thoroughly reviewed. Two independent neurologists (SL and JAR) measured patients’ GOSs. If the GOSs measured by the two neurologists did not match, a consensus was reached after discussion. CT protocol for SAH patients was the same as the previous study [4]. Initial brain CT and CT angiography were performed within 12 h from the onset of SAH. All CT studies were performed using 64-channel scanners (Light Speed VCT, GE Healthcare, Milwaukee, WI, USA) [4]. Investigators who were blinded to clinical information evaluated each patient’s CT scans using a commercial image-viewing software (Centricity RA1000 PACS Viewer, GE Healthcare) [4]. Initial Glasgow Coma Scale (GCS) was defined as GCS at admission. Vasospasm was diagnosed when the mean velocity of transcranial Doppler was higher than 120 cm/sec [14]. Systemic hemodynamic variations were evaluated using Lindegaard ratio [20]. Vasospasm was also diagnosed via follow-up CT angiography and digital subtraction angiography. DCI was diagnosed as decreased level of consciousness or development of new focal neurological deficits lasting for at least 60 min after eliminating other factors (e.g., hydrocephalus, seizure, or systemic complications) [8, 14].

Initial CRP levels were measured within 12 h after admission. The day on admission was defined as day 1. CRP data on days 1 to 7 were collected. Serum CRP levels were measured using immunoturbidimetric assays (CRPL3, Roche Diagnostics, Indianapolis, IN, USA) with a lower reference limit of 0.3 mg/dL [21]. The maximal level of CRP (CRP_Max_) was defined as the peak level from days 1 through 4. Subsequent CRP (CRP_min_) level was determined as the minimal level from days 5 to 7. CRP kinetics was expressed as CRP clearance (CRPc) calculated as the percentage of ΔPCT (the difference between CRP_Max_ and subsequent measurement) over CRP_Max_ (100x[CRP_Max_ - CRP_min_]/CRP_Max_) [21].

### Statistical analyses

A “Clinical Data Warehouse Darwin-C” was designed for investigators to search and retrieve de-identified medical records from the electronic archive system. After finalizing the patient list for this study, laboratory data were extracted from the Clinical Data Warehouse Darwin-C. The clinical data were collected by a trained study coordinator using a standardized case report form. All data are presented as means ± standard deviations (SD) for continuous variables and numbers (percentages) for categorical variables. Log-transformations were used to compare CRPc data due to their skewed distribution. Data were compared using Student’s *t*-test for continuous variables and Chi-square test or Fisher’s exact test for categorical variables. Pearson’s correlation coefficient (*r*) was calculated to determine the correlation between CRP and severity scores. Predictive performances of CRPs and CRPc were assessed using areas under the curve (AUCs) of receiver operating characteristic (ROC) curves for sensitivity vs. 1-specificity. AUCs were compared using the nonparametric approach published by DeLong et al. [22] for two correlated AUCs. The optimal cut-off for CRP to predict poor neurological outcomes was obtained using the ROC curve and the Youden index [23, 24]. Variables with *P* values less than 0.2 in univariate analyses and clinically relevant variables were subjected to a stepwise multiple logistic regression model to obtain statistically meaningful predictor variables. Adequacy of the prediction model was also determined using the Hosmer-Lemeshow test. All tests were two-sided. *P* < 0.05 was considered statistically significant. All data analyses were performed using IBM SPSS version 20 (IBM, Armonk, NY, USA).

## Results

### Baseline characteristics

The mean age of all SAH patients was 59.6 ± 13.2 years. There were 51 (32.7%) male patients. Hypertension (38.5%) was the most common comorbidity. Sixty-five (41.4%) patients had WFNS grade 4 or 5. The WFNS grade was higher in the group with poor neurological outcome than in those with favorable neurological outcome (4.2 ± 1.1 vs. 2.0 ± 1.4, *P* < 0.001). Baseline characteristics of enrolled patients are presented in Table 1. The most common locations of ruptured aneurysms were the anterior communicating artery (26.3%) and the middle cerebral artery (23.1%). However, 10 (6.4%) patients had no aneurysm or ruptured aneurysm. The aneurysm was treated within 72 h in most patients (87.8%). Endovascular coiling was performed in 94 (60.3%) patients and surgical clipping was carried out in 44 (28.2%) patients.

**Table 1:** Baseline characteristics

	Favorable neurological outcome (*n* = 109)	Poor neurological outcome (*n* = 47)	*P* value
Age (yr) — mean ± SD	56.6 ± 12.5	66.4 ± 12.2	< 0.001
Gender, male — no. of patients (%)	37 (33.9)	14 (29.8)	0.748
BMI (kg/m^2^) — mean ± SD	23.6 ± 3.3	23.1 ± 5.2	0.576
Comorbidities — no. of patients (%)			
Hypertension	36 (33.0)	24 (51.1)	0.052
Current smoker	25 (22.9)	7 (14.9)	0.355
Diabetes mellitus	5 (4.6)	5 (10.6)	0.289
Dyslipidemia	6 (5.5)	0 (0)	0.235
Previous TIA or stroke	3 (2.8)	2 (4.3)	0.999
Malignancy	2 (1.8)	2 (4.3)	0.745
Chronic kidney disease	3 (2.8)	0 (0)	0.608
Ischemic heart disease	2 (1.8)	0 (0)	0.874
Hunt and Hess Classification — no. of patients (%)			< 0.001
2	69 (63.3)	2 (4.3)	
3	20 (18.3)	7 (14.9)	
4	13 (11.9)	11 (23.4)	
5	7 (6.4)	27 (57.4)	
Glasgow Coma Scale — mean ± SD	12.8 ± 3.5	7.1 ± 3.8	< 0.001
Modified Fisher classification — no. of patients (%)			< 0.001
1	25 (22.9)	1 (2.1)	
2	8 (7.3)	0 (0)	
3	57 (52.3)	23 (48.9)	
4	19 (17.4)	23 (48.9)	
Pupil reactivity — no. of patients (%)			< 0.001
Both intact pupil reflex	100 (91.7)	28 (59.6)	
One unreactive pupil	3 (2.8)	3 (6.4)	
Both unreactive pupils	6 (5.5)	16 (34.0)	
Aneurysm location — no. of patients (%)			0.036
Anterior communicating artery	33 (30.3)	8 (17.0)	
Anterior cerebral artery & distal	3 (2.8)	7 (14.9)	
Middle cerebral artery & distal	24 (22.0)	12 (25.5)	
Internal carotid artery	6 (5.5)	7 (14.9)	
Posterior communicating artery	20 (18.3)	6 (12.8)	
Posterior circulation	12 (11.0)	3 (6.4)	
No aneurysm	7 (6.4)	3 (6.4)	
Unknown	4 (3.7)	1 (2.1)	
Hydrocephalus — no. of patients (%)	48 (44.0)	34 (72.3)	0.002
Intraventricular hemorrhage — no. of patients (%)	25 (22.9)	23 (48.9)	0.002

*SD,* standard deviation; *BMI,* body mass index; *TIA,* transient ischemic attack

### Clinical outcomes

Among 44 patients undergoing surgical clipping, 14 (9.0%) manifested poor neurological outcomes. DCI was accompanied in 31 (19.9%) patients. Treatment characteristics of SAH patients are presented in Table 2. Among 145 (92.9%) survivors, 109 (69.9%) showed favorable neurological outcomes (GOS of 4, or 5, Fig. 1). In addition, among 65 (41.7%) patients with poor-grade SAH, 54 survived to discharge (83.1%) and 26 (40.0%) showed favorable neurological outcomes.

**Table 2:** Treatment characteristics

	Favorable neurological outcome (n = 109)	Poor neurological outcome (n = 47)	*P* value
Aneurysm treatment and timing — no. of patients (%)			0.001
Early treatment within 72 h	100 (91.7)	37 (78.7)	
Early but non-aneurysm detection	1 (0.9)	1 (2.1)	
Late treatment	1 (0.9)	8 (17.0)	
No treatment	7 (6.4)	1 (2.1)	
Aneurysm management — no. of patients (%)			
Coiling	70 (64.2)	24 (51.1)	0.173
Surgical clipping	30 (27.5)	14 (29.8)	0.925
Endotracheal intubation during over 24 h	14 (12.8)	34 (72.3)	< 0.001
External ventricular drainage — no. of patients (%)	36 (33.0)	28 (59.6)	0.004
Vasospasm — no. of patients (%)	46 (42.2)	18 (38.3)	0.781
Delayed cerebral ischemia — no. of patients (%)	17 (15.6)	14 (29.8)	0.069
Decompressive craniectomy — no. of patients (%)	1 (0.9)	9 (19.1)	< 0.001
Barbiturate coma therapy — no. of patients (%)	2 (1.8)	3 (6.4)	0.325

### CRPs and CRP clearance

**Fig. 2:**
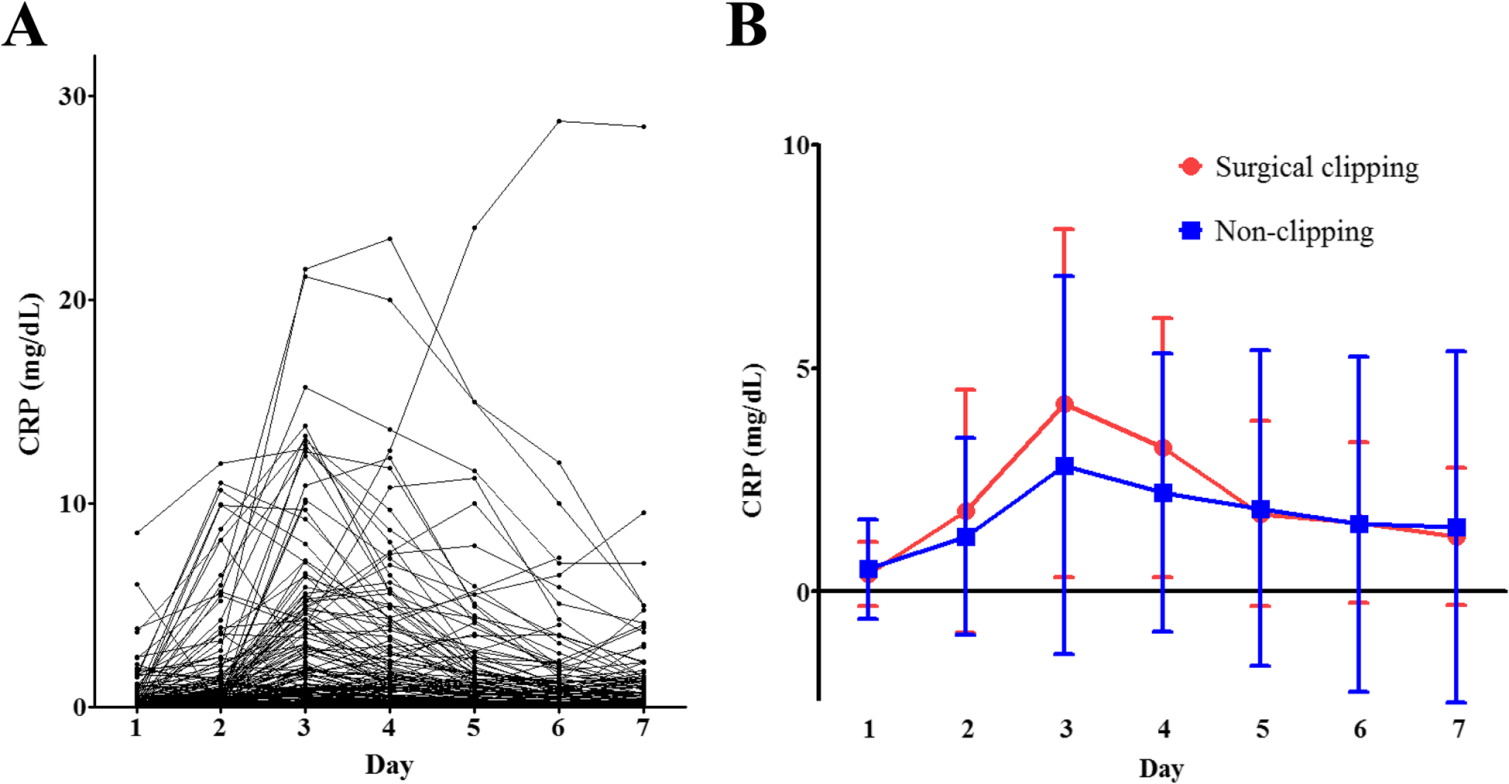
Daily changes of serum C-reactive protein (CRP) levels in all patients (**a**). CRP levels (mean ± standard deviation) with or without surgical clipping (**b**)

**Fig. 3:**
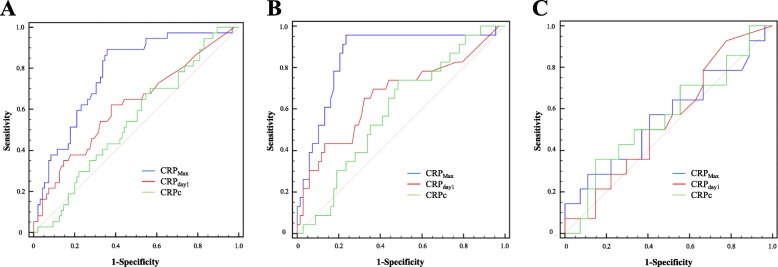
Receiver operating characteristic curves for prediction of poor outcomes using CRP and its modification. The area under the curve of CRP_Max_ (C-statistic: 0.778, 95% confidence interval [CI]: 0.698–0.846) was greater than that of CRP_day1_ or clearance of CRP (CRPc) in all patients (*P* = 0.007, *P* < 0.001, respectively) (**a**). The C-statistic of CRP_Max_ in patients without surgical clipping (C-statistic: 0.856, 95% CI: 0.767–0.921) was greater than that of all SAH patients (**b**). However, the performance of CRP_Max_ in patients with clipping (C-statistic: 0.562, 95% CI: 0.399–0.716) was very poor (**c**). CRP, C-reactive protein; CRP_day1_, CRP level on admission; CRP_Max_, maximal level of CRP within four days; CRPc, clearance of CRPs

**Fig. 4:**
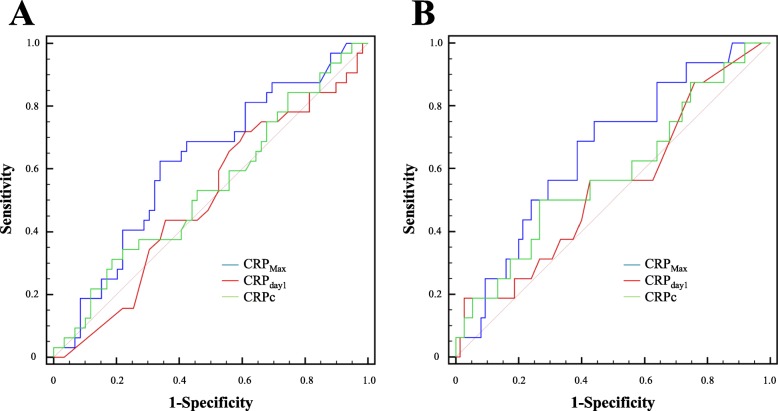
Receiver operating characteristic curves used to predict vasospasm (**a**) and delayed cerebral ischemia (**b**) in patients without surgical clipping. CRP, C-reactive protein; CRP_day1_, CRP level on admission; CRP_Max_, maximal level of CRP within four days; CRPc, clearance of CRPs

A progressive increase in CRP levels was detected from the admission until the third or fourth day, followed by a slow decline toward the seventh day. Daily changes in CRP levels are shown in Fig. 2. In this study, the CRP_day1_ and CRP_Max_ values in the poor neurological outcome group were significantly higher than in those with favorable outcome (0.9 ± 1.6 vs. 0.3 ± 0.5, *P* = 0.022 and 6.2 ± 5.3 vs. 2.2 ± 3.0, *P* < 0.001, respectively). However, CRPc did not differ significantly between those with favorable and poor neurological outcomes (4.3 ± 0.4 vs. 4.3 ± 0.4, *P* = 0.785). In ROC curve analysis associated with the prediction of poor neurological outcomes, the AUC of CRP_Max_ was greater than that of CRP_day1_ or CRPc (*P* = 0.007, *P* < 0.001, respectively, Fig. 3a). Therefore, the performance of CRP_Max_ for outcome prediction was significantly better than that of CRP_day1_ or CRPc. The C-statistic of CRP_Max_ was 0.778 (95% confidence interval [CI]: 0.698 to 0.846) in all SAH patients. Although the AUC of CRP_Max_ in patients without surgical clipping (C-statistic: 0.856, 95% CI: 0.767–0.921) was greater than that of all SAH patients (Fig. 3b), the performance of CRP_Max_ in patients undergoing surgical clipping (C-statistic: 0.562, 95% CI: 0.399–0.716) was very poor (Fig. 3c). A cut-off of CRP_Max_ > 2.1 mg/dL showed a sensitivity of 78.8% (95% CI: 61.1 to 91.0%) and a specificity of 82.3% (95% CI: 72.1 to 90.0%) in patients without surgical clipping. In ROC curve analysis for the prediction of vasospasm (Fig. 4a) and DCI (Fig. 4b) in patients without surgical clipping, the C-statistics of CRP_Max_ were 0.619 (95% CI: 0.512 to 0.719) and 0.658 (95% CI: 0.551–0.754), respectively.

The effect of CRP on the neurological outcome differed by surgical clipping. The interaction between CRP_Max_ and surgical clipping was associated with poor neurological outcome (*P* = 0.047) (Table 3). Based on simple correlation analysis, the CRP_Max_ of patients without surgical clipping showed a linear correlation with Hunt-Hess grade and WFNS grade (*r* = 0.643, *P* < 0.001 and *r* = 0.598, *P* < 0.001, respectively). Multivariable logistic regression analysis revealed that age (adjusted odd ratio [OR]: 1.10, 95% CI: 1.052–1.158), initial GCS (adjusted OR: 0.74, 95% CI: 0.647–0.837), and CRP_Max_ without surgical clipping (adjusted OR: 1.27, 95% CI: 1.066–1.516) were significantly associated with poor neurological outcomes in SAH patients (Hosmer-Lemeshow Chi-squared = 7.92, *df* = 8, *P* = 0.442). However, CRP_Max_ with surgical clipping (adjusted OR: 1.01, 95% CI: 0.840–1.223) was not associated with poor neurological outcomes in SAH patients.

**Table 3:** Multivariable logistic regression of clinically relevant variables associated with poor neurological outcomes

	*B*	OR (95% CI)	*P* value
Age	0.009	1.10 (1.052–1.158)	< 0.001
Initial Glasgow Coma Scale	−0.307	0.74 (0.647–0.837)	< 0.001
Interaction of CRP_Max_ × surgical clipping	−0.248	0.78 (0.611–0.997)	0.047
Surgical clipping	1.662	5.27 (1.091–25.442)	0.039
CRP_Max_	0.240	1.27 (1.066–1.516)	0.008

*CRP*_*Max*_ maximal level of C-reactive protein within four days

## Discussion

The study evaluated whether CRP levels or changes in their levels can be used to predict neurological outcomes of SAH patients. The major study findings are as follows. Patients with poor-grade SAH manifested considerable survival rate (83.1%) and favorable neurological prognoses (40.0%). The CRP levels increased progressively from admission day 1 to day 3 or 4, followed by a slow decline by day 7. Initial and maximal CRP levels within four days in the poor neurological outcome group were significantly greater than in the group with favorable outcome. The maximal CRP levels within four days were more strongly associated with poor neurological outcomes than the initial CRP levels on admission. However, changes in CRP levels were not correlated with neurological outcomes of SAH patients. We found that the effect of CRP on the neurological outcome differed by surgical clipping. Although the maximal CRP levels within four days facilitate the prediction of neurological outcomes of SAH patients without surgical clipping, they were not associated with neurological outcomes in SAH patients who underwent surgical clipping. The CRP in patients without surgical clipping showed a linear correlation with Hunt-Hess grade and WFNS grade. Therefore, higher levels of CRP as an inflammatory marker may increase the severity of SAH. Multivariable logistic regression analysis revealed that age, initial GCS, maximal CRP levels, and the interaction between maximal CRP and surgical clipping were significantly associated with poor neurological outcomes of SAH patients.

Traditionally, CRP is the most frequently used biomarker to diagnose sepsis or infection [21, 25]. CRP is a sensitive and nonspecific inflammatory marker in acute phase of inflammation and infection [8]. The level of CRP increases up to 1000-fold within a few hours in response to inflammatory stimulation such as trauma, burn, cancer, and various infections or inflammatory diseases [8, 26, 27]. In addition, the level of CRP may peak at 48 h after the initial stimulus and decline to baseline within 7 to 12 days after the inflammatory stimulus disappears [8, 26, 27].

CRP is associated with several cerebrovascular diseases and cardiovascular diseases [9–11, 17]. Elevated CRP levels are significantly associated with poor neurological outcomes in patients with ischemic stroke [9, 10, 17]. Ischemic injury resulting from cerebral arterial occlusion manifests as acute local inflammation and changes in the levels of inflammatory cytokines [10]. In addition, the CRP measurement facilitates accurate prevention and appropriate treatment of coronary thrombotic events [11, 13]. Due to the similar vascular pathophysiology of DCI and acute myocardial infarction or ischemic stroke, the serum CRP levels might have a prognostic value in acute phase after SAH [8, 9, 11, 13]. Therefore, the serum CRP levels may be correlated with the severity of SAH as well as with poor neurological outcomes [11].

DCI is a complex, multifactorial syndrome associated with vasospasm, early brain injury, cortical spreading depression, and microthrombosis [1, 5–7]. Additionally, extravasated blood and early activated inflammatory pathways post-SAH trigger a cascade of reactions involving the release of pro-inflammatory and vasoactive factors [5]. Activation of the systemic immune response characterized by elevated levels of circulating cytokines results in cerebrovascular vasospasm [15]. Therefore, elevated CRP levels are linked to vasospasm and DCI after SAH [11, 13–15]. However, this study revealed minimal association of CRP levels with vasospasm and DCI. These associations may facilitate the accurate diagnosis of vasospasm and DCI because multimodal monitoring or routine angiography was not performed in all patients with poor-grade SAH in this study.

CRP as a continuous variable is associated with clinical prognosis [8, 13, 26]. Since CRP can increase rapidly after ruptured aneurysm, determination of only a single absolute CRP level may not reflect the severity of disease at a very early stage. In ischemic stroke, several studies reported a variation in the time points of CRP measurement for prognosis [9, 10, 17, 18]. Therefore, a serial follow-up of CRP levels rather than CRP levels at a single time point may facilitate prognostic prediction. Eventually, daily CRP measurements conducted during at least four days after SAH may be useful in predicting the outcomes. Additionally, the CRP measurements are inexpensive, readily available, consistent, and reproducible in most countries [21]. Daily measurements of CRP levels may also facilitate the monitoring of the course of severe sepsis in patients [21]. Changes in CRP concentrations are correlated with outcomes of severe septic patients [21]. However, they were not associated with neurological outcomes of SAH patients in this study.

In this study, surgical clipping was a confounding factor, which affected the prediction of neurological outcomes of SAH patients in this study. Trauma or surgical stress also increases the CRP levels [8]. Therefore, elevated CRP levels related to surgical clipping might skew the prognosis of clinical outcomes. In this study, the measurement of CRP was not significantly associated with poor neurological outcomes in SAH patients who underwent surgical clipping. Eventually, in SAH patients without surgical clipping, the maximal CRP levels within four days were significantly associated with poor neurological outcomes.

This study has several limitations. First, it was a retrospective review of medical records. A few data of CRP values were missing. The GOS was also retrospectively determined based on medical records. However, a consensus following discussion by two independent specialists might have attenuated the bias partially. Second, the non-randomized nature of the registry data might have resulted in selection bias. Third, vasospasm was mostly diagnosed by transcranial Doppler rather than via CT angiography or digital subtraction angiography. Although transcranial Doppler is a non-invasive monitoring tool for cerebral vasospasm, it has a limited diagnostic ability [28]. Additionally, the diagnosis of DCI was retrospectively established based on medical records. Brain magnetic resonance imaging was not used routinely to detect silent cerebral infarction after SAH, which may have limited the detection of vasospasm and DCI in this study. Fourth, the CRP levels might be increased in patients with chronic inflammatory diseases and neurodegenerative diseases [29]. Although we excluded patients with severely neurodegenerative diseases and chronic neurological abnormality, we could not exclude patients with mild or undiagnosed illness. Finally, our study has limited statistical power due to its small sample size. Prospective large-scale studies are needed to confirm the usefulness of early serial measurement of CRP in predicting neurological outcomes of patients with SAH to obtain evidence-based conclusions.

## Conclusions

Early serial measurements of CRP might be useful in predicting neurological outcomes of SAH patients without surgical clipping. Furthermore, maximal CRP levels within four days after SAH are significantly associated with poor neurological outcomes in these patients.

## Data Availability

Regarding data availability, our data are available at the Harvard Dataverse Network (10.7910/DVN/KEZNDR).

## Abbreviations

CRP: C-reactive protein
CRP_day1_: CRP level on admission
CRP_Max_: Maximal level of CRP within four days
CRPc: Clearance of CRPs
CT: Computed tomography
DCI: Delayed cerebral ischemia
GOS: Glasgow Outcome Scale
ICU: Intensive care unit
SAH: Subarachnoid hemorrhage
WFNS: World Federation of Neurosurgical Societies
